# Activation of HIV Transcription by the Viral Tat Protein Requires a Demethylation Step Mediated by Lysine-specific Demethylase 1 (LSD1/KDM1)

**DOI:** 10.1371/journal.ppat.1002184

**Published:** 2011-08-18

**Authors:** Naoki Sakane, Hye-Sook Kwon, Sara Pagans, Katrin Kaehlcke, Yasuhiro Mizusawa, Masafumi Kamada, Kara G. Lassen, Jonathan Chan, Warner C. Greene, Martina Schnoelzer, Melanie Ott

**Affiliations:** 1 Gladstone Institute of Virology and Immunology, University of California, San Francisco, California, United States of America; 2 Pharmaceutical Frontier Research Laboratory, Yokohama, Japan; 3 Department of Medicine, University of California, San Francisco, United States of America; 4 Department of Microbiology and Immunology, University of California, San Francisco, United States of America; 5 Functional Proteome Analysis, German Cancer Research Center (DKFZ), Heidelberg, Germany; Fred Hutchinson Cancer Research Center, United States of America

## Abstract

The essential transactivator function of the HIV Tat protein is regulated by multiple posttranslational modifications. Although individual modifications are well characterized, their crosstalk and dynamics of occurrence during the HIV transcription cycle remain unclear.

We examine interactions between two critical modifications within the RNA-binding domain of Tat: monomethylation of lysine 51 (K51) mediated by Set7/9/KMT7, an early event in the Tat transactivation cycle that strengthens the interaction of Tat with TAR RNA, and acetylation of lysine 50 (K50) mediated by p300/KAT3B, a later process that dissociates the complex formed by Tat, TAR RNA and the cyclin T1 subunit of the positive transcription elongation factor b (P-TEFb). We find K51 monomethylation inhibited in synthetic Tat peptides carrying an acetyl group at K50 while acetylation can occur in methylated peptides, albeit at a reduced rate. To examine whether Tat is subject to sequential monomethylation and acetylation in cells, we performed mass spectrometry on immunoprecipitated Tat proteins and generated new modification-specific Tat antibodies against monomethylated/acetylated Tat. No bimodified Tat protein was detected in cells pointing to a demethylation step during the Tat transactivation cycle. We identify lysine-specific demethylase 1 (LSD1/KDM1) as a Tat K51-specific demethylase, which is required for the activation of HIV transcription in latently infected T cells. LSD1/KDM1 and its cofactor CoREST associates with the HIV promoter *in vivo* and activate Tat transcriptional activity in a K51-dependent manner. In addition, small hairpin RNAs directed against LSD1/KDM1 or inhibition of its activity with the monoamine oxidase inhibitor phenelzine suppresses the activation of HIV transcription in latently infected T cells.

Our data support the model that a LSD1/KDM1/CoREST complex, normally known as a transcriptional suppressor, acts as a novel activator of HIV transcription through demethylation of K51 in Tat. Small molecule inhibitors of LSD1/KDM1 show therapeutic promise by enforcing HIV latency in infected T cells.

## Author Summary

One of the remaining questions in HIV research is how the virus establishes a dormant (latent) stage and thereby escapes eradication by current antiretroviral therapy. Latently infected T cells do not produce significant amounts of viral genomes or viral proteins due to the silencing of a specific step in the viral life cycle called transcription. Viral transcription can be reactivated in latently infected cells, a process that rekindles HIV infection after antiretroviral therapy is discontinued. A key regulator of viral transcription is the viral Tat protein. Here we identify a novel cellular enzyme that regulates HIV transcription through the modification of the Tat protein. This enzyme, LSD1, is generally known as a transcriptional suppressor. In HIV infection, however, it acts as a transcriptional activator because downregulation of LSD1 expression or inhibition of its enzymatic activity suppresses reactivation of HIV from latency. Our findings provide novel insight into the mechanisms of HIV latency and identify a potential new strategy that may help to keep HIV dormant in latently infected cells.

## Introduction

Epigenetic processes are critical in the regulation of gene expression from the integrated HIV provirus and have become a focal point of research in therapeutics for HIV latency. Latently infected T cells persist in HIV-infected individuals despite highly active antiretroviral therapy (HAART) and rekindle the infection when HAART is discontinued [1], [2]. In the majority of latently infected cells, HIV infection is blocked at the transcriptional level. Therapeutic efforts are aimed at permanently silencing HIV gene expression in latently infected cells or at “flushing out” the viral reservoirs by reverting the transcriptional silencing that lies at the core of HIV proviral latency.

Known epigenetic processes involved in the regulation of HIV gene expression include DNA methylation [3], [4], chromatin remodeling events [5], [6], [7], posttranslational modifications of histones [8], [9] and posttranslational modifications of the HIV Tat protein [10], [11], [12], [13], [14], [15], [16]. Tat is an essential viral gene product that potently activates HIV gene expression through its unique interactions with the TAR element located at the 5′ ends of nascent viral transcripts and the cellular positive transcription elongation factor b (P-TEFb) [17], [18]. Two Tat species naturally exist in HIV-infected cells: a full-length Tat protein of ∼101 aa length encoded by both *tat* exons and a shorter splice variant of 72 aa length encoded by the first *tat* exon. Both Tat forms are transcriptionally active and form a trimolecular complex with the cyclin T1 subunit of P-TEFb and TAR RNA to recruit the kinase activity of CDK9 to elongating HIV transcripts. The bulk of Tat is produced after successful integration of the provirus into the host genome where it activates its own production via a feed-forward mechanism [19].

Several posttranslational modifications of Tat have been identified that modulate the interactions of Tat with P-TEFb and TAR RNA [20] (see Table S1). Two of these modifications, acetylation of K50 and monomethylation of K51, occur at adjacent residues within the arginine-rich motif (ARM) in Tat, a region involved in TAR RNA binding, nuclear localization and protein stability [21]. K50 is the preferred target for the acetyltransferase activity of p300/KAT3B in Tat while K51 is monomethylated by the lysine methyltransferase Set7/9/KMT7 [10], [11], [16], [22]. K50 and K51 are also targets of the acetyltransferase activity of hGCN5/KAT2A and the di- or trimethyltransferase activity of SETDB1/KMT1 [15], [22].

K50 acetylation and K51 monomethylation have both important positive regulatory functions in Tat transactivation. Monomethylation of K51 strengthens the interactions of Tat with P-TEFb and TAR RNA while acetylation of K50 dissociates the Tat/TAR/P-TEFb complex and recruits the PCAF/KAT2B histone acetyltransferase to the elongating RNA polymerase II complex [16], [23], [24], [25]. These findings form the basis for a dynamic view of the Tat transactivation cycle in which changes in the modification status of Tat occur sequentially and govern differential cofactor interactions of a single Tat molecule during HIV transcription [26], [27].

We were intrigued by the close proximity of the two modifications in Tat (K50 acetylation and K51 methylation) and speculated that a bimodified protein may exist in cells. Similar studies were previously performed with the p53 tumor suppressor protein and supported the model that lysines in close proximity to each other are sequentially methylated and acetylated [28], [29]. However, we did not detect bimodified Tat in cells using mass spectrometry or newly generated antibodies specific for monomethylated/acetylated Tat. Instead, we identified LSD1/KDM1 as a Tat demethylase and an unexpected new transcriptional coactivator required for activation of HIV gene expression in latently infected T cells.

## Results

### Acetylation of K50 inhibits monomethylation of K51 in Tat *in vitro*

**Figure 1:**
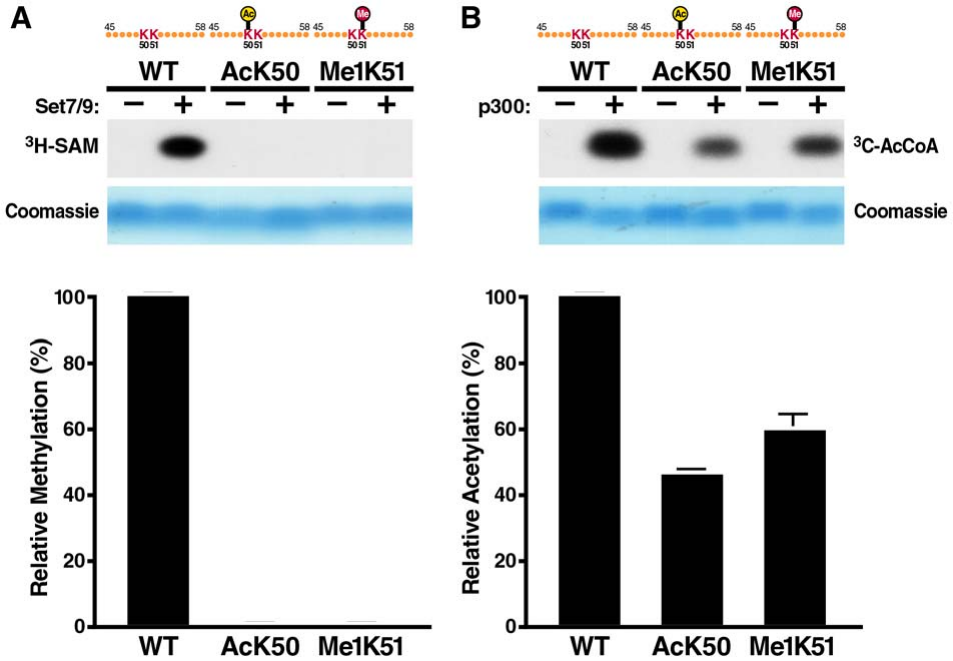
*In vitro* acetylation and methylation assays using synthetic Tat peptides. (A) *In vitro* methylation assays of Tat ARM peptides (aa 45–58). Unmodified, K50-acetylated or K51-methylated peptides were incubated with recombinant SET7/9/KMT7 and ^3^H-radiolabeled S-adenosyl-L-methionine (SAM). Peptides were separated by Tris-Tricine gel electrophoresis and visualized by autoradiography. A quantification of band intensities of three independent experiments is shown below. (B) *In vitro* acetylation assays of Tat ARM peptides. Unmodified, K50-acetylated or K51-methylated peptides were incubated with recombinant p300-HAT and ^14^C-acetyl coenzyme A. Peptides were processed as in A. A quantification of band intensities of three independent experiments is shown below.

To examine how acetylation of K50 affects monomethylation of the neighboring K51 residue, we incubated short synthetic Tat peptides (aa 48–58) carrying an acetylated lysine at position 50 with recombinant Set7/9/KMT7 enzyme and radiolabeled S-adenosyl-L-methionine (SAM). Reactions were dissolved on a high percentage Tris-Tricine gel and examined by autoradiography. Acetylation at K50 completely suppressed methylation of the peptide by Set7/9/KMT7 (Figure 1A). The same was observed when a K51-monomethylated peptide was tested in the reaction indicating that K50 is not a target of the Set7/9/KMT7 monomethyltransferase activity (Figure 1A). We also performed the inverse experiment and incubated a Tat peptide carrying a monomethyl group at position 51 with the K50 acetyltransferase p300/KAT3B and observed that acetylation can proceed, albeit with a 40% decrease in efficiency as compared to an unmodified peptide (Figure 1B). Interestingly, a K50-acetylated peptide was further acetylated by p300/KAT3B confirming previous results that K51 also functions as a target of the p300/KAT3B acetyltransferase activity, especially when K50 is not available [10], [11], [30]. Similar results were observed when the reactions were performed with full-length synthetic Tat proteins (aa 1–72) carrying acetylated K50 or monomethylated K51 residues (Figure S1). These data demonstrate that *in vitro* monomethylation cannot occur efficiently on an acetylated Tat substrate supporting previous data that point to a role of K51 monomethylation early in the Tat transactivation cycle before acetylation of K50 [16]. Because we find that K50 acetylation can occur *in vitro* when Tat is monomethylated, we examined whether Tat is subject to sequential monomethylation/acetylation *in vivo*.

### Mass spectrometry of immunoprecipitated Tat

**Figure 2:**
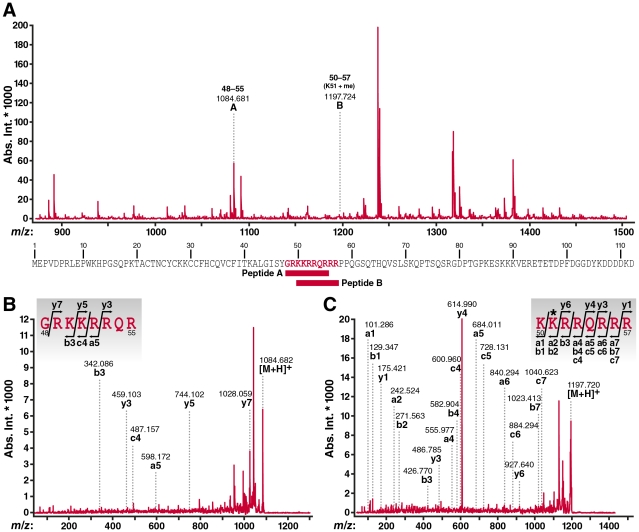
MALDI-TOF mass spectrometric analysis of cellular Tat confirms K51 monomethylation. (A) MALDI-TOF MS spectrum of digested peptides from Tat-FLAG (900-1,500* m/z*) immunoprecipitated from J-Lat A2 cells activated with TNFα. The peptide ions (designated as A and B) further analyzed by MS/MS are indicated by *m/z* values and number of amino acid sequence. Their position within the Tat-FLAG molecule used in this study is indicated below. Please note that studies were performed with chymotrypsin to avoid restraints in trypsin-based cutting caused by modifications of lysines. However, some identified ARM peptides are shorter than anticipated based on the predicted size of chymotrypsin-digested peptides in Tat which is likely due to a contamination with some trypsin-like activity in the chymotrypsin preparations used in this experiment. (B) MALDI-TOF/TOF MS/MS spectra of peptide A ion (1084.681 *m/z*). (C) MALDI-TOF/TOF MS/MS spectra of peptide B ion (1197.724 *m/z*). In these spectra, identified fragment ions were denoted by the ion types, a, b, c, y according to the nomenclature by Roepstorff and Fohlman [57]. The assignment of fragment ions to the amino acid sequence of the ARM region of the Tat molecule was inserted in each spectrum. The symbol * indicates methylated amino acid residue.

To search for monomethylated/acetylated Tat in cells, we performed mass spectrometry of Tat immunoprecipitated from TNFα-activated J-Lat A2 cells. This Jurkat-derived cell line harbors an integrated bicistronic lentiviral vector, which expresses FLAG-tagged Tat and GFP from the integrated HIV LTR (LTR-Tat-IRES-GFP) upon stimulation with TNFα [31]. Immunoprecipitated material was separated by SDS-PAGE and stained with FLAMINGO fluorescence dye. The Tat band was cut from the gel and applied to in-gel digestion with chymotrypsin. Residual digested peptides were analyzed by MALDI-TOF/TOF mass spectrometry. A representative MALDI-TOF MS spectrum of the digested peptides is shown in Figure 2A, and more than 100 peptide ion signals were detected. A peptide encompassing the Tat ARM region without modification was detected at 1084.681 *m/z*, which was identified as the peptide from glycine 48 to arginine 55 in the Tat-FLAG molecule by MALDI-TOF/TOF MS/MS analysis (Figure 2B). We also detected a mass signal at 1197.724 *m/z*, which corresponded to the Tat peptide from lysine 50 to arginine 57 carrying a monomethyl group at lysine 51 (Figure 2C). Bimodified Tat (AcK50/Me1K51) was not detected in this experiment. In addition, we did not detect dimethylation at K51, but detected a peptide in which both K50 and K51 carried a mass addition of 42 Da, indicating that these residues could be either acetylated or trimethylated in cells (data not shown). Mass spectrometry cannot differentiate efficiently between these two modifications. We previously confirmed that acetylation of K50 exists in cells using acetylation-specific Tat antibodies [25] but could not detect trimethylation of K51 using trimethyl-Tat-specific antibodies [32] supporting a model where both residues may be acetylated rather than trimethylated in cells. Further experiments using modification-specific antibodies directed against both sites are currently underway.

### Bimodified Tat is not detected in cells

**Figure 3:**
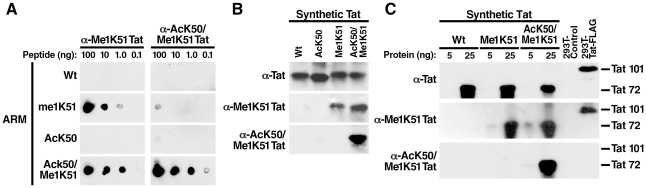
No detection of cellular acetylated/methylated Tat by newly generated Tat antibodies. (A) Dot-blot analysis of ARM peptides using α-Me1K51 or α-AcK50/Me1K51 Tat antibodies. (B) Western blot analysis of synthetic Tat (aa 1–72) with α-Tat, α-Me1K51 Tat or α-AcK50/Me1K51 Tat antibodies. Of note, we have not succeeded so far to synthesize a Tat peptide of 101 aa length corresponding to the full-length Tat species encoded by two *tat* exons in cells. (C) Whole cell lysates from 293T cell transfected with FLAG-tagged Tat (aa 1–101) were subjected to immunoprecipitation with α-FLAG agarose. Purified proteins were analyzed by western blot analysis together with synthetic Tat proteins using the indicated antibodies.

To independently analyze the existence of bimodified (AcK50/Me1K51) Tat in cells, we generated a polyclonal antiserum specific for doubly modified Tat. ARM peptides carrying an acetyl group at position 50 and a monomethyl group at position 51 were injected into rabbits and affinity purified on a column carrying the bimodified antigen. The resulting antibodies (α-AcK50/Me1K51 Tat) were specific for the bimodified ARM peptides and did not react with singly modified peptides in dot blot analysis (Figure 3A). In contrast, an antiserum that we previously generated against monomethylated K51 in Tat (α-Me1K51 Tat) [16] reacted with ARM peptides monomethylated at K51 as expected but also showed cross-reactivity with bimodified peptides (Figure 3A). The same results were obtained when we tested the antibodies by western blot analysis of synthetic Tat proteins (aa 1–72), which carried either one or both modifications. The α-AcK50/Me1K51 Tat antibodies specifically recognized doubly modified Tat while the α-Me1K51 Tat recognized both methylated and doubly modified Tat (Figure 3B). No cross-reactivity was observed with unmodified or acetylated Tat.

To test whether doubly modified Tat exists in cells, we transfected FLAG-tagged Tat into 293T cells and purified Tat with α-FLAG agarose. K51 methylated Tat was readily detected by western blot analysis using α-Me1K51 Tat antibodies while no signal was detected with the α-AcK50/Me1K51 Tat antibodies (Figure 3C). Of note, both antibodies recognized their cognate antigens with similar sensitivities as shown by western blot analysis of full-length synthetic methylated and acetylated/methylated Tat proteins (Figure 3C). Similar experiments were performed with antibodies against AcK50Me2K51 and AcK50Me3K51 in Tat and showed no reactivity with Tat in cells (data not shown). This result confirms the data obtained by mass spectrometry, which indicate that doubly modified Tat is not a major Tat species in cells.

### LSD1/KDM1 demethylates Tat at K51

**Figure 4:**
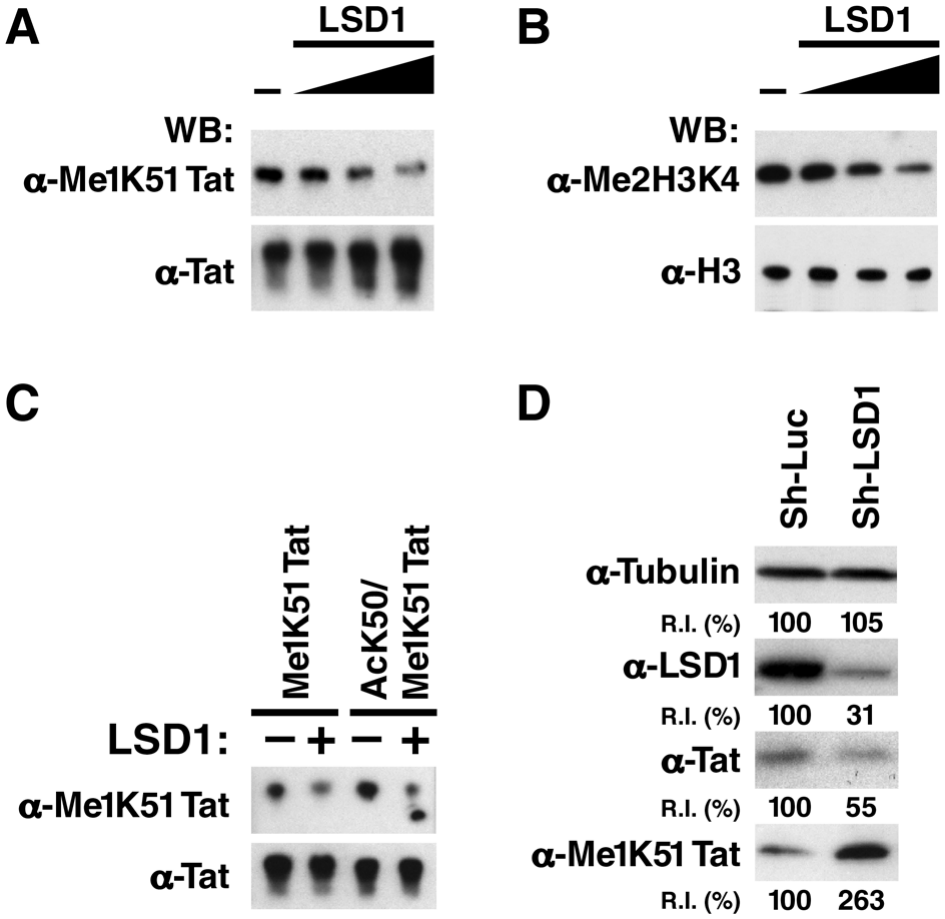
LSD1/KDM1 demethylates mono-methylated K51 in Tat. (A) Synthetic K51-mono-methylated Tat proteins were incubated with increasing amounts of recombinant LSD1/KDM1 (0, 0.5, 1, 2 µg) for 1 h at 37°C. Reaction products were analyzed by western blotting using α-Tat, α-Me1K51 Tat antibodies. (B) Purified histone proteins were subjected to the same procedure as in A and analyzed by α-Me2H3K4 or α-histone H3 antibodies. (C) Synthetic K51-mono-methylated Tat or K50-acetylated/K51-monomethylated Tat proteins were incubated with 1 µg of recombinant LSD1 as described in A. (D) Increase in K51-monomethylation of Tat in LSD1 shRNA-infected J-Lat A2 cells. Whole cell lysates isolated from J-Lat A2 cells infected with shRNAs directed against LSD1 or control shRNAs and stimulated with TNFα were analyzed by western blotting with indicated antibodies. Band intensities were quantified using ImageJ Software (NIH). R.I.: Relative intensities of bands as compared to the control-vector transduced cells (100%).

We speculated that Tat is demethylated at K51 before acetylation occurs. Recombinant LSD1/KDM1 demethylated synthetic monomethylated Tat in a dose-dependent manner as shown by western blot analysis using α-Me1K51 Tat antibodies (Figure 4A). LSD1/KDM1 also demethylated its cognate substrate, dimethyl lysine 4 in histone H3, as expected (Figure 4B). Interestingly, LSD1/KDM1 demethylated monomethylated Tat *in vitro* regardless of whether the neighboring K50 residue was acetylated or not, suggesting that LSD1/KDM1 may demethylate Tat in cells either before or immediately after acetylation had occurred (Figure 4C).

To test whether LSD1/KDM1 is involved in the demethylation of Tat in cells, we introduced lentiviral vectors carrying shRNAs against LSD1/KDM1 into J-Lat A2 cells and reduced endogenous expression of LSD1/KDM1 (Figure 4D). We then induced expression of Tat with TNFα and monitored monomethylation of Tat K51 using western blotting with α-Me1K51 Tat antibodies. Monomethylation of Tat was 2.6-fold enhanced in cells expressing shRNAs against LSD1/KDM1 as compared to cells expressing control shRNAs although the overall expression of Tat was reduced (Figure 4D). This reduction is explained by the negative effect of LSD1-knockdown on Tat transcriptional activity (see below), which drives Tat expression from the LTR in these cells. Collectively, these results demonstrate that LSD1/KDM1 demethylates Tat K51 *in vitro* and in cells.

### LSD1/KDM1 associates with the HIV promoter *in vivo*

**Figure 5:**
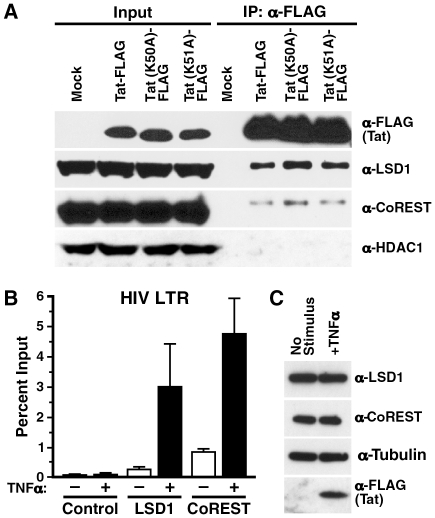
*In vivo* recruitment of LSD1 and CoREST to the HIV LTR. (A) Co-immunoprecipitation of endogenous LSD1 and CoREST with Tat/FLAG and the Tat K51A mutant in transiently transfected 293T cells. (B) Chromatin immunoprecipitation analysis of LSD1, CoREST and HDAC1 in J-Lat A2 cells. A2 cells were stimulated with TNFα over night and chromatin immunoprecipitation was performed using α-LSD1, α-CoREST, α-HDAC1 or no antibodies followed by real-time RT-PCR with primers specific for the HIV LTR region. (C) Whole cell lysates isolated from A2 cells stimulated with TNFα over night were analyzed by western blotting using α-LSD1, α-tubulin or α-FLAG antibodies.

To test whether LSD1/KDM1 interacts with Tat in cells, FLAG-tagged Tat proteins were expressed in 293T cells after transient transfection. Following immunoprecipitation with α-FLAG antibodies, endogenous LSD1/KDM1 was detected by western blotting in the immunoprecipitated material (Figure 5A). Tat proteins carrying point mutations either in K50 (K50A) or K51 (K51A) also efficiently coimmunoprecipitated with LSD1/KDM1 indicating that the interaction was not dependent on demethylation of K51 in Tat. A similar, albeit weaker interaction was observed when we tested Tat's interaction with the LSD1/KDM1 cofactor CoREST [33], [34] suggesting that Tat may recruit a functional LSD1/KDM1/CoREST complex to the HIV promoter. In contrast, no interaction of Tat or Tat mutants was observed with cellular HDAC1, often also described as part of LSD1/KDM1/CoREST corepressor complexes [35], [36], [33], [37]. It is not clear at the moment whether the observed interactions of Tat with LSD1/KDM1 or CoREST are direct or mediated by other cellular proteins.

To test the hypothesis that LSD1/KDM1 and CoREST are recruited to the HIV LTR, we performed chromatin immunoprecipitation assays. Chromatin was prepared from J-Lat A2 cells, in which Tat expression was stimulated by TNFα treatment or which were left nonstimulated. Quantitative PCR analysis of the immunoprecipitated material with primers specific for the HIV LTR indicated that LSD1/KDM1 and CoREST, while only present at low concentrations at the promoter under nonstimulated conditions, were specifically recruited in response to TNFα stimulation (Figure 5B, LSD1 and CoREST). No signal was detected when immunoprecipitation was performed with beads alone (Figure 5B, Control). Overall cellular expression of LSD1/KDM1 and CoREST was unchanged in response to treatment with TNFα in J-Lat A2 cells (Figure 5C). These results demonstrate that LSD1/KDM1 and CoREST are recruited to the HIV LTR in response to Tat. However, recruitment may also occur indirectly via other LTR activators in response to TNFα treatment.

### LSD1/KDM1 acts as an activator of HIV transcription

**Figure 6:**
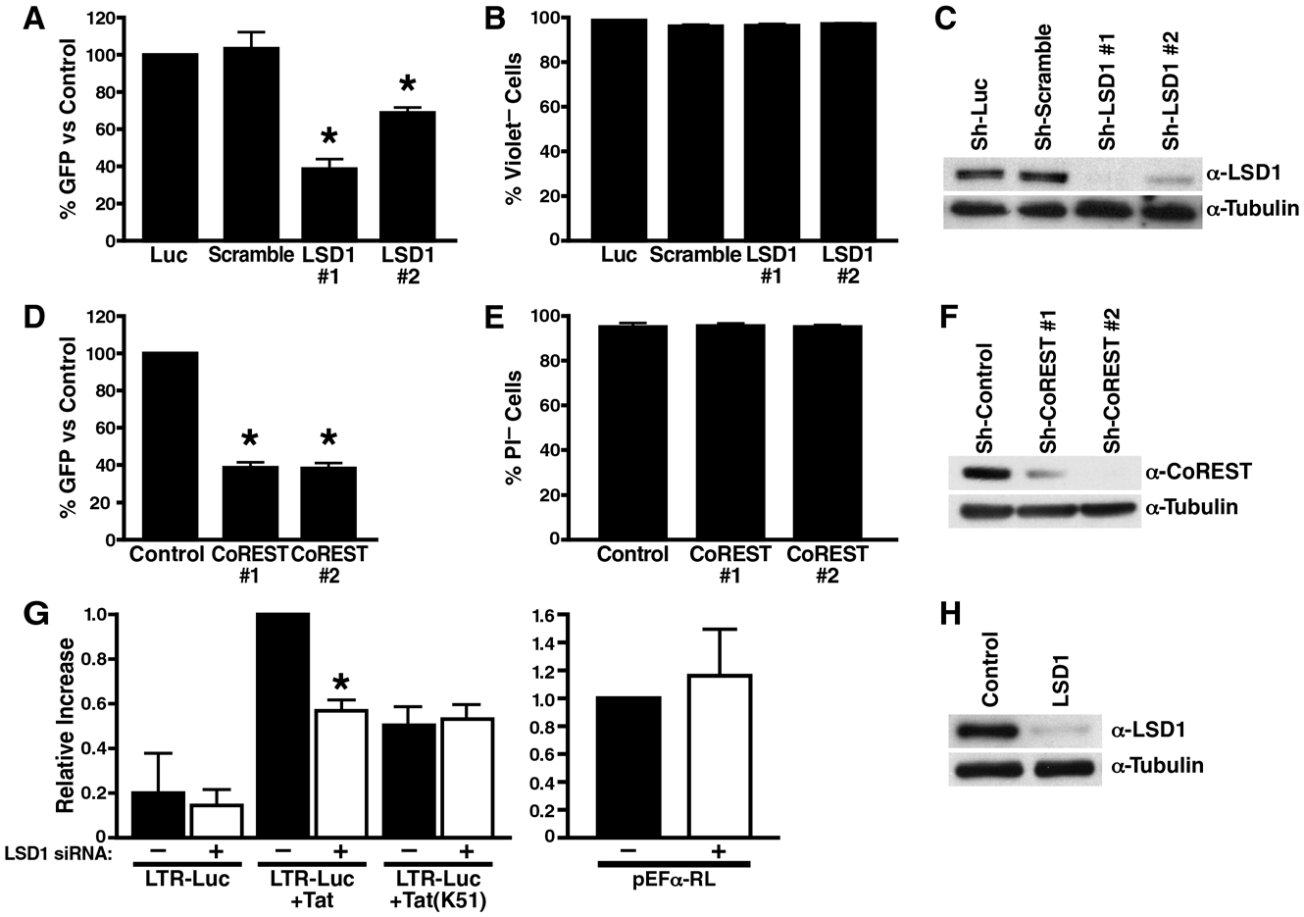
LSD1/KDM1 and CoREST activate HIV transcription. (A) Lentiviral vectors expressing shRNAs against LSD1 (#1 and #2), luciferase or scrambled shRNAs were infected into J-Lat A2 cells for 5–10 days; cells were then stimulated with a low dose of TNFα (0.08 ng/ml) over night. Number of GFP+ cells was analyzed by flow cytometry and expressed as percent GFP+ cells as compared to luciferase-shRNA-infected A2 cells. The average (mean±SEM) of three independent experiments is shown. *corresponds to a p value <0.01. (B) Number of violet dye-negative cells was measured as a marker of cell viability by flow cytometry in cells described in (A). Of note, propidium iodide (PI) staining could not be performed in these cells because of the mCherry marker expressed by the LSD1 shRNA vector (C) Whole cell lysates from shRNA-expressing cells were analyzed by western blotting using α-LSD1 and α-tubulin antibodies. (D) Lentiviral vectors expressing shRNAs against CoREST (#1 and #2) or control shRNAs were infected into J-Lat A2 cells. The same experiment as in (A) was performed. The average (mean±SEM) of three independent experiments is shown. *corresponds to a p value <0.01. (E) Number of PI-negative cells was measured as a marker of cell viability by flow cytometry in cells described in (D). (F) Whole cell lysates from shRNA-infected A2 cells were analyzed by western blotting using α-CoREST and α-tubulin antibodies. (G) SiRNA-transfected HeLa cells (48 h after siRNA transfection) were re-transfected with an HIV LTR luciferase reporter construct and expression vectors for wildtype or K51A mutant Tat. In parallel, same amounts of EF-1α RL reporter constructs as used for Tat were transfected to monitor Tat expression. Luciferase or Renilla activities were measured 24 h after plasmid transfections. The relative differences in luciferase activity as compared to wildtype Tat transactivation were calculated. The average (mean±SEM) of three independent experiments is shown. *corresponds to a p value <0.01. (H) Whole cell lysates from siRNA-transfected HeLa cells were analyzed by western blotting using α-LSD1 and α-tubulin antibodies.

To test the function of LSD1/KDM1 in HIV transcription, A2 cells were transduced with lentiviral vectors expressing two different shRNAs directed against LSD1/KDM1 or control shRNAs directed against firefly luciferase or a scrambled shRNA. All vectors also expressed the mCherry marker to track infection efficiencies. More than 90% of cells expressed mCherry after lentiviral vector infection, and no difference in infection efficiencies was observed between the different lentiviral vectors (not shown). ShRNA-expressing cells were stimulated with TNFα, and expression of GFP was measured by flow cytometry. GFP expression was reduced by 40-60% in LSD1/KDM1 knockdown cells as compared to cell lines expressing luciferase or scrambled shRNAs (Figure 6A). No toxicity of LSD1 knockdown was observed in shRNA-treated cells as measured by dye exclusion in flow cytometry (Figure 6B). ShRNA#1 had a stronger suppressive effect on GFP expression than shRNA#2 mirroring the degree of LSD1/KDM1 knockdown in these cells (Figure 6C). The same result was obtained in 5A8 J-Lat cells harboring a full-length GFP-tagged latent HIV genome (Figure S2). Here, reactivation from latency is achieved by stimulation with α-CD3/CD28 antibodies in ∼40% of cells. Only 5 or 15% of cells reactivate HIV transcription in cells treated with LSD1#1 or LSD1#2 shRNAs, respectively, confirming that LSD1 is important for full transcriptional activity after reactivation from latency after T cell receptor stimulation (Figure S2). A similar suppression of GFP expression in the absence of cell toxicity was observed in A2 cells, in which the expression of CoREST was downregulated (Figure 6D–F). Interestingly, in A72 cells, in which GFP expression is driven by the LTR alone in the absence of Tat, no effect of either downregulation of cellular LSD1 or CoREST expression was observed pointing to a specific effect of LSD1/KDM1 and CoREST in Tat transactivation (Figure S3). Collectively, these results demonstrate that an LSD1/KDM1/CoREST complex, often a suppressor of cellular gene expression, functions as a co-activator of HIV transcription.

To test whether LSD1/KDM1 activates HIV transcription through Tat demethylation, we introduced siRNAs specific for LSD1/KDM1 or control siRNAs into HeLa cells. Cells were then co-transfected with the HIV LTR luciferase reporter gene and an expression construct for Tat. Tat transactivation of the HIV LTR was suppressed by ∼50% when expression of LSD1/KDM1 was reduced in cells indicating that LSD1/KDM1 is a positive cofactor of Tat transactivation (Figure 6G). Expression of the TatK51A mutant resulted in a similar decrease in Tat transactivation (∼50%) as previously reported [16], but no further reduction was observed in LSD1/KDM1 knockdown cells supporting the model that LSD1/KDM1 activates Tat transactivation through K51 demethylation (Figure 6G). The transcriptional activity of the HIV LTR alone was also reduced in LSD1/KDM1 knockdown cells (∼28%) although values did not reach statistical significance indicating that an additional target for LSD1/KDM1 may or may not exist at the HIV LTR in the absence of Tat (Figure 6G). Importantly, LSD1/KDM1 knockdown had no suppressive effect on the EF-1α promoter that was driving Tat expression in these co-transfection experiments excluding the possibility that LSD1/KDM1 controls Tat expression and not Tat function (Figure 6G). Successful knockdown of LSD1/KDM1 expression was confirmed by western blotting (Figure 6H).

### Phenelzine suppresses reactivation of HIV gene expression from latency

**Figure 7:**
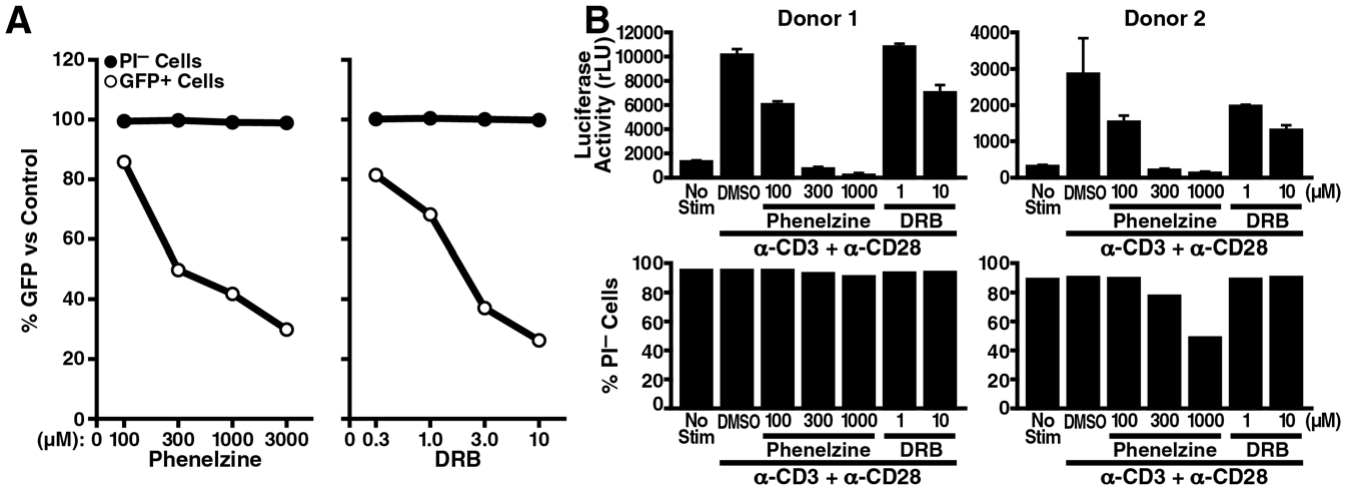
LSD1/KDM1 as a potential drug target in HIV transcription. (A) J-Lat A2 cells were stimulated with 0.08 ng/ml of TNFα in the presence of increasing amounts of phenelzine (100, 300, 1000 or 3000 µM), DRB (0.3, 1, 3 or 10 µM) or DMSO as carrier control overnight. Numbers of GFP+ cells (marker of HIV LTR activity) as well as propidium iodide (PI)-negative cells (marker of cell viability) were analyzed by flow cytometry. The percentage compared to DMSO-treated control cells was calculated. The average of two independent experiments is shown. (B) Purified resting primary CD4+ T cells were infected at a high m.o.i. with infectious HIV-NL4-3 luciferase reporter virus. Three days after infection, cells were stimulated with α-CD3 and α-CD28 antibodies in the presence of phenelzine (100, 300, or 1000 µM), DRB (1 or 10 µM) or DMSO overnight followed by analysis for luciferase activity and PI uptake.

Since LSD1/KDM1 belongs to the amine oxidase enzyme superfamily that oxidatively removes methyl groups from di- or monomethylated lysines, some monoamine oxidase (MAO) inhibitors can act as LSD1/KDM1 inhibitors [34], [38], [39], [40]. It was recently reported that the MAO antidepressant agent phenelzine (phenethylhydrazine) is far more potent in inhibiting LSD1/KDM1 activity in cells than previously appreciated [38]. To test the activity of this agent in HIV infection, J-Lat A2 cells were treated with increasing amounts of phenelzine or the CDK inhibitor 5, 6-dichloro-1-β-D-ribofuranosyl-1H-benzimidazole (DRB), a known Tat inhibitor. Phenelzine, similar to DRB, prevented TNFα-mediated activation of gene expression in a dose-dependent manner, albeit at ∼150 fold higher concentrations than DRB (IC_50_ = 300 µM Figure 7A, white circle). No cell toxicity was observed for both agents at the tested concentrations (Figure 7A, black circle).

The same experiment was performed in a primary T cell model of HIV latency. Quiescent CD4+ T cells were isolated from blood of two healthy donors and were spin-inoculated with an infectious clone of HIV expressing luciferase within the *nef* open reading frame following a similar protocol as previously described [41]. Infected CD4+ cells were cultured with the integrase inhibitor saquinavir for 3 days to ensure that postintegration latency was measured and then treated with increasing amounts of phenelzine followed by stimulation with α-CD3 and α-CD28 antibodies to activate latent HIV transcription. Activation of luciferase expression was successfully suppressed by phenelzine treatment confirming the effectiveness of the drug in the context of a full-length infectious clone of HIV in primary T cells (Figure 7B). A slight decrease in cell viability was observed in one donor at the highest concentration of phenelzine (Figure 7B). However, no effect of phenelzine on cell viability was observed in additional two donors, in whom reactivation from HIV latency were also successfully inhibited by the drug (Figure S4). Interestingly, in activated primary T cells, phenelzine was more efficient in suppressing HIV gene expression than DRB while in latently infected, but not activated, cells phenelzine, contrary to DRB, had no suppressive effect on luciferase expression (Figure S5). Collectively, these results identify phenelzine as a potent new inhibitor of HIV reactivation from latency and support the model that LSD1/KDM1 is a novel activator of HIV transcription through Tat demethylation.

## Discussion

We investigated whether Tat lysine methylation and acetylation events within the Tat ARM are linked via a demethylation step mediated by LSD1/KDM1. Similar to previous reports on the tumor suppressor p53 [28], [29], we find that methylation and acetylation of Tat can occur sequentially *in vitro* with methylation at K51 occurring first allowing subsequent acetylation of K50, albeit at diminished efficiency. Detailed *in vivo* analysis of the Tat ARM reveals that a bimodified Tat form does likely not exist in cells because it was not detected by mass spectrometry and by western blotting using newly generated bispecific Tat antibodies. Instead, we identify LSD1/KDM1 as a K51-specific Tat demethylase and a novel transcriptional activator of HIV transcription. These findings may be clinically relevant because we demonstrate that phenelzine, a MAO inhibitor with activity against LSD1/KDM1, successfully suppresses re-activation of HIV transcription in latently infected T cells.

Until recently, it was unclear whether methylation of lysines is reversible. Today, there exist two types of lysine demethylases, LSD1/KDM1 and Jumoji C domain-containing demethylases [42]. LSD1/KDM1 is a flavin adenine dinucleotide (FAD)-dependent amine oxidase, which can demethylate lysine 4 in histone H3 (an activatory mark) and lysine 9 in histone H3 (a silencing mark). LSD1/KDM1 can remove methyl groups from mono- or di-, but not tri-methylated lysines [43], [44]. Since the FAD-dependent amine oxidase family of enzymes, which includes MAO-A, MAO-B and LSD1/KDM1, share a common mechanism for the oxidative cleavage of the unactivated nitrogen, known MAO inhibitors such as phenelzine used in this study or others have activity against LSD1/KDM1 [34], [38], [39], [40].

It was recently reported that besides histones, LSD1/KDM1 can also demethylate non-histone proteins including the tumor suppressor p53, the DNA methylase Dnmt1, and transcription factor E2F1 [45], [46], [47], [48]. Demethylation of p53 by LSD1 alters the interaction of p53 with its coactivator 53BP1 and represses the proapoptotic function of p53 [45]. Similarly, demethylation of Dnmt1 by LSD1 triggers a loss of protein stability and a loss of global DNA methylation while demethylation of E2F1 is required for E2F1 stabilization and apoptotic function [46], [47]. Our finding that Tat function is activated by LSD1/KDM1-mediated demethylation adds another nonhistone protein to the growing list of LSD1/KDM1 substrates. Like E2F1, Tat is activated by LSD1/KDM1 demethylation, a finding that supports the model that the coordinated occurrence of Tat modifications is essential for efficient Tat transcriptional activity.

Our finding that LSD1/KDM1 and CoREST are both recruited to the activated HIV LTR *in vivo* points to Tat demethylation as a novel mechanism how HIV may corrupt the function of a known corepressor complex to enhance its own replication. The interaction with CoREST is known to direct the LSD1/KDM1 activity towards lysine 4 in nucleosomal histone H3 and is associated with transcriptional repression [33], [34]. However, LSD1/KDM1 can also act as a transcriptional activator for instance through demethylation of lysine 9 in histone H3 in conjunction with androgen receptor-mediated transcription [44], through demethylation of lysine 9 in histone H3 in α-herpesvirus infections [49] or through demethylation of the E2F1 transcription factor which activates its apoptotic function [46]. Evidence that support a role of Tat demethylation in the LSD1/KDM1 coactivator function in HIV transcription comes from the Tat K51A mutant, which remains unaffected by siRNA-mediated downregulation of LSD1/KDM1 expression. However, other LSD1 substrates may exist at the HIV promoter that activate HIV transcription when Tat is absent. An attractive target is methylated lysine 9 in histone H3 at the HIV provirus, which was previously linked to HIV latency [9], [50], [51] and is the target of the activatory function of LSD1/KDM1 in the transcriptional control of herpes simplex virus- and varicella zoster virus latency [49]. Notably, a recent study shows that the latter function also involves CoREST indicating that a functional LSD1/KDM1/CoREST complex can function as suppressor of cellular gene expression or as activator of viral transcription [52]. Interestingly, HDAC1/2 are generally part of this complex, but we do not observe any interaction of Tat with HDAC1 in our study. This may point to HDAC2 or another HDAC associated with the LSD1/KDM1 subcomplex recruited to the HIV LTR or may indicate that Tat specifically dissociates HDACs from LSD1/KDM1 complexes involved in its demethylation. Notably, binding of CoREST, but not HDAC1, to LSD1/KDM1 restored the ability of recombinant LSD1 to demethylate nucleosomal substrates while HDACs are thought to act upstream of LSD1/KDM1 by providing hypoacetylated substrates for demethylation [33].

**Figure 8:**
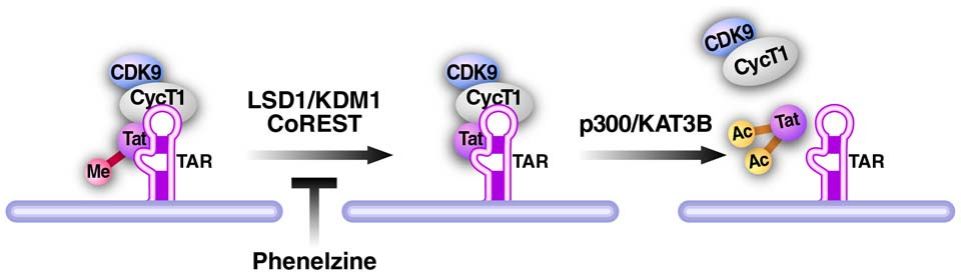
Model of LSD1/KDM1 action in HIV transcription. Demethylation of Tat by the LSD1/KDM1/CoREST complex is a required new step during Tat transactivation of the HIV LTR. We propose that it may occur before acetylation of Tat by p300/KAT3B. Inhibition of LSD1/KDM1 by phenelzine blocks this demethylation step and inhibits Tat transactivation during reactivation from latency.

We focused here on the interaction between Tat demethylation at K51 and K50 acetylation, but interplay may also exist between K51 demethylation and other known Tat modifications such as arginine methylation within the Tat ARM (Table S1). We have previously shown that both acetylation and deacetylation of K50 in Tat are required for full Tat transactivation. While acetylation of K50 by p300/KAT3B dissociates Tat from TAR RNA and P-TEFb, deacetylation by SIRT1 may be necessary to recycle nonacetylated Tat for reentry into the transactivation cycle [12]. Here, we show that the same “Yang/Yang” principle applies to methylation and demethylation of K51 in Tat. Both, methylation of K51 by Set7/9/KMT7 and demethylation of K51 by LSD1/KDM1 activate Tat transactivation because knockdown or inhibition of each enzyme leads to reduction of Tat transcriptional activity in a K51-dependent manner. We propose a model where demethylation of Tat occurs as a critical step during the Tat transactivation cycle possibly before acetylation of Tat by p300/KAT3B occurs (Figure 8). In support of this model, we have previously shown that monomethylation of Tat is an early event that strengthens the interaction of Tat with TAR RNA and P-TEFb [16]. In addition, we show here that *in vitro* monomethylation at K51 decreases efficient acetylation of K50 by p300/KAT3B supporting the model that prior demethylation is required to allow full Tat acetylation at K50 and possibly at K51 in cells. We also found in preliminary experiments that LSD1/KDM1 coimmunoprecipitates with p300/KAT3B in cellular extracts pointing to a potential recruiting function of LSD1/KDM1 for p300/KAT3B to Tat (N. Sakane and M.Ott, unpublished data).

Our data provide important first evidence that LSD1 inhibitors may function as therapeutics to suppress reactivation of HIV transcription in latently infected cells. They further support the model that targeting Tat posttranslational modifications may be a valid therapeutic strategy to control HIV transcription and latency; Tat becomes “locked” in one modified state when individual modifying enzymes are blocked and the normal flow of Tat modifications is disturbed. Interestingly, MAO inhibitors with inhibitory functions towards LSD1/KDM1 have suppressive activity in latent infections of α-herpesvirus [49]. Our results indicate that they may have a broader antiviral application that includes HIV-1. The development of more specific LSD1/KDM1 inhibitors will bring further validation to the model that LSD1/KDM1 is an important new drug target in the treatment of latent HIV infection.

## Methods

### Cells, reagents and antibodies

HeLa and 293T cells (obtained from the American Type Culture Collection), and the J-Lat clone A2 [31] were maintained under standard cell-culture conditions. The following antibodies were commercially available: α-LSD1/KDM1 (#ab51877, abcam, Cambridge, MA), α-CoREST (#ab24166, abcam), α-FLAG M2 (#F-3165 Sigma-Aldrich, St. Louis MO), α-histone H3K4me2 (#07-030, Millipore, Billerica, MA), α-histone H3 (#07-690, Millipore), α-tubulin (#T6074, Sigma-Aldrich), α-Tat (MMS-116P, Covance, Emeryville, CA), and α-CD28 (#16-0289-85 eBioscience, San Diego CA). α-K51 monomethylated Tat polyclonal antibodies were previously described [16]. α-CD3 (OKT-3) was obtained from the UCSF monoclonal antibodies core facility. The α-HDAC1 polyclonal antibodies were a kind gift of Eric Verdin, Gladstone Institute of Virology and Immunology, San Francisco.

Phenelzine Sulfate was purchased from Spectrum Chemical MHG Corp. (#3032 Gardena, CA) and Enzo Life Sciences (#EI-217, Plymouth meeting, PA). Saquinavir was obtained from the AIDS Research and Reference Reagent Program, Division of AIDS, NIAID, NIH. Recombinant human TNFα was purchased from Humanzyme (#HZ-1014, Chicago, IL).

The synthetic Tat proteins (aa 1–72) was synthesized as previously described [12] together with the Tat ARM short peptide (aa 45-58) by Dr. Hans-Richard Rackwitz (Peptide Specialty Laboratories GmbH, Heidelberg, Germany).

The HIV LTR luciferase construct, the EF-1α-Tat/FLAG expression vector, the K51A mutated EF-1α-Tat/FLAG expression vector and the pEF-1α-RL (Renilla luciferase) were described before [16]. The His-tagged LSD1 prokaryotic expression vector was previously described (Department of Pathology, Harvard Medical School, [43]).

A modified version of the pSicoR lentiviral vector that encodes the mCherry reporter gene driven by an EF-1α promoter (pSicoRMS) [53], [54] was kindly provided by Matthew Spindler (Gladstone Institute of Cardiovascular Disease). ShRNAs targeting LSD1 (LSD1 #1:GAAGGCTCTTCTAGCAATA, LSD1 #2: CATGTGCCTGTTTCTGCCATG) were cloned into pSicoRMS. The pSicoRMS containing a non-targeting control sequence (shScramble: GTCAAGTCTCACTTGCGTC) [55] and targeting luciferase (shLuciferase: CTTACGCTGAGTACTTCGA) was kindly provided by Dr. Silke Wissing (Gladstone Institute of Virology and Immunology). ShRNAs against CoREST and control empty pLKO.1 vector were purchased from Thermo Fischer Scientific (Waltham MA).

### In-gel digestion and MALDI-TOF mass spectrometric analysis of Tat protein

C-terminal FLAG-tagged Tat protein (Tat/FLAG) purified from J-Lat A2 cells (∼100 ng) was further purified by SDS-PAGE (FLAMINGO gel stain, Bio-Rad Hercules, CA). Tat band was excised and washed with 200 µL of 50 mM ammonium bicarbonate containing 50% (v/v) ethanol followed by 200 µL of ethanol twice. The Tat protein in the gel was reduced with 10 mM DTT in 50 mM ammonium bicarbonate for 1 h at 56°C and alkylated with 55 mM iodoacetamide in 50 mM ammonium bicarbonate for 30 min at room temperature. After reduction and alkylation, the gel was dehydrated with acetonitrile 3 times. The gel was rehydrated by adding 200 µL of 50 mM ammonium bicarbonate with 5 ng/µL of chymotrypsin (Roche, Penzberg, Upper Bavaria, Germany) and incubated at 30°C for 2 h. Digested Tat peptides were extracted from the gel with 1% (v/v) formic acid containing 30% (v/v) acetonitrile followed by 1% (v/v) formic acid containing 60% (v/v) acetonitrile. The extracted peptide solution was dried up by speed vac. Then, residual peptides were reconstituted with 30 µL of 0.1% (v/v) TFA containing 2% (v/v) acetonitrile and desalted by ZipTip_C18_ (Millipore) according to the manufacturer's description. 2 µL of cleaned peptide solution eluted from ZipTip_C18_ was deposited on the Bruker metallic MALDI target (MTP384 ground steel, Bruker Daltonics, Billerica, MA) and mixed with 2 µL of saturated matrix solution (α-cyano-4-hydroxycinnamic acid solution in 33% (v/v) acetonitrile, 0.1% (v/v) TFA). Peptide mixture was allowed to dry at room temperature. The peptide mixture was analyzed by ultraflex III TOF/TOF (Bruker Daltonics) MALDI-TOF/TOF mass spectrometer, operated in reflector mode for positive ion detection, and controlled by flexControl 3.0 software. For MS/MS acquisitions, the ions of interest were fragmented by laser-induced decay, and mass of fragments was analyzed using LIFT mode. Monoisotopic mass was determined using flexAnalysis 3.0 software with the SNAP peak picking algorithm. The modifications of peptides were analyzed using UniMod database in the Biotools software.

### Generation of bimodified Tat antibodies

The strategy to generate bimodified Tat (AcK50/Me1K51) specific antibodies was performed as previously described [16], [32]. Briefly, KLH conjugated bimodified ARM peptides (AcK50/Me1K51) were injected into rabbits. The same peptides were used for affinity purification of the resulting antiserum. Specificity of antibodies was monitored by dot-blot analysis using ARM peptides and western blot analysis using synthetic full-length Tat proteins.

### *In vitro* enzymatic assays

Protein expression and purification of recombinant LSD1 and *in vitro* demethylation reactions of LSD1 (0.5–2.0 µg) with synthetic Tat proteins (3 µg) or purified total cellular histones (8 µg) were performed as previously described [43]. The reactions were analyzed by western blotting using α-Me1K51 Tat antibodies (1 µg ml). *In vitro* methylation reactions with synthetic Tat protein (aa 1-72; 1 µg) and ARM peptides (aa 45-58; 100 µM), Set7/9-KMT7 enzyme (2 µg Millipore), and ^3^H-S-Adenosyl Methionine (Perkin Elmer) were performed as previously described [16]. *In vitro* acetylation reactions with synthetic Tat proteins (1 µg), ARM peptides (100 µM), GST-p300 HAT enzyme (aa 1195-1810; 5 µg; [56]) and ^14^C-acetyl CoA (0.1 µCi; Perkin Elmer) were performed as described [10]. Reactions were separated by SDS-PAGE or Tris-Tricine gel electrophoresis and visualized by autoradiography.

### RNAi experiments

siRNA analysis for HeLa cells were performed as described [16]. Briefly, HeLa cells were transfected with pooled LSD1 and control siRNAs (200 pmol, Dharmacon; Lafayette, CO) using Oligofectamine (#58303, Invitrogen, Carlsbad, CA) and were retransfected after 48 h with the HIV LTR luciferase construct (200 ng), Tat-expressing vectors (2 ng), and corresponding amounts of the empty vector using lipofectamine reagent (#50470, Invitrogen). Cells were harvested 24 h later and processed for luciferase assays (Luciferase Assay System, #E1501, Promega, Madison, WI) or western blotting.

J-Lat A2 cells were transduced with pseudotyped pSicoRMS-derived lentiviral vectors expressing shRNAs against LSD1 (shLSD1 #1 and #2), against luciferase (shLuciferase) or a nontargeting shRNA control (ShScramble). These lentiviral vectors also express the mCherry protein under the control of the EF-1α promoter (see cells, reagents and antibodies). 5 to 10 days after infection, cells were treated with 0.08 ng/ml of TNFα for 12 h. Expression of GFP and mCherry was analyzed by flow cytometry (BD LSRII, Beckton Dickinson, Franklin Lakes, NJ). Similar experiments were performed using pLKO.1-derived vectors expressing shRNAs against CoREST (shCoREST #1 and #2) or empty vector controls (shControl). When pLKO.1 vectors were used, puromycin was added one day after shRNA infection (1 ng/ml). Cell viability was determined by propidium iodide staining (#P-3566, Invitrogen) or LIVE/DEAD Fixable Violet Dead Cell Stain Kit (#L34958, Invitrogen) followed by flow cytometry.

### Chromatin immunoprecipitation experiments

Chromatin immunoprecipitations from J-Lat A2 cells were performed as previously described [4], [16]. Chromatin solutions were isolated from A2 cells treated with TNFα (2 ng/ml) and were immunoprecipitated with α-LSD1 antibodies (abcam), α-CoREST antibodies (abcam) or control rabbit pre-immune serum. The immunoprecipitated material was quantified by real-time PCR with primers specific for the HIV LTR using the ABI7700 Sequence Detection System (Applied Biosystems, Foster City, CA) and the 2x Hot Sybr real-time PCR kit (#HSM-400, McLab, South San Francisco, CA). Primer sequences were: HIV LTR upstream: GAGCCCTCAGATCCTGCATA, HIV LTR downstream: AGCTCCTCTGGTTTCCCTTT.

### Co-immunoprecipitation experiments

293T cells were transfected with Tat expressing vector using Fugene 6 reagent (Roche). 24 h after transfection, cells were lysed in IP buffer (250 mM NaCl, 0.1% NP40, 20 mM NaH_2_PO_4_ (pH 7.5), 5 mM EDTA, 30 mM sodium pyrophosphate, 10 mM NaF and protease inhibitors) and immunoprecipitated with α-FLAG M2 agarose (Sigma-Aldrich) over night at 4°C. Beads were extensively washed and analyzed by western blotting with α-LSD1, α-CoREST, α-HDAC1 or monoclonal α-FLAG antibodies.

### Primary T cell model of HIV latency [41]

The infectious NL4-3-luciferase clone of HIV was generated by cloning the BamHI to XhoI fragment of pNL-Luc-E^-^R^-^ within the nef coding region into pNL4-3 [48]. This generates a fully infectious clone capable of multiple rounds of infection and producing luciferase driven from the LTR promoter. Infectious particles were produced after transfection of the clone into 293T cells. Two days after transfection, the transfected supernatants were collected and concentrated by ultra-centrifuge (20,000 rpm, 2 h), and virus concentration was determined by analyzing concentration of p24^gag^ (HIV-1 antigen p24 ELISA kit #NEK050A001KT, Perkin Elmer). CD4+ T cells were isolated from human whole blood buffy coats obtained from anonymous donors by centrifugation onto a Histopaque-1077 cushion (#10771, Sigma-Aldrich), enrichment of T cells by rosetting with sheep red blood cells (#CS115 Colorado Serum, Denver, CO) and depletion of non-CD4+ T cells with the CD4+ T cell isolation kit (#130-091-155, Miltenyi Biotec Bergisch Gladbach, Germany) and AutoMACS cell separator (Miltenyi Biotec). Purity of isolated CD4+T cells was confirmed by flow cytometry. For infection, 1 µg of p24^gag^ was used for 5×10^6^ CD4 T cells. The mixture of virus and cells were centrifuged at 2400 rpm for 2 h. After spinoculation, cells were cultured in the presence of 5 µM saquinavir for 3 days and were then stimulated with α-CD3 (2.5 µg/ml, coated) and α-CD28 antibodies (1 µg/ml, soluble) in the presence or absence of phenelzine (100 µM–1 mM). After over night incubation, cells were harvested and processed for luciferase assays (Luciferase Assay System, Promega). Cell viability was determined by propidium iodide staining (#P-3566, Invitrogen).

## Supporting Information

Figure S1 **Interplay between Tat monomethylation at K51 and acetylation at K50 using synthetic Tat proteins (aa 1–72).** (A) Radioactive *in vitro* methylation assays of synthetic Tat and K50-acetylated synthetic Tat incubated with recombinant SET7/9/KMT7 and ^3^H-radiolabeled S-adenosyl-L-methionine (SAM). Reaction products were separated by SDS-PAGE and visualized by autoradiography. (B) Radioactive *in vitro* acetylation assays of synthetic Tat and K51-methylated synthetic Tat incubated with recombinant p300-HAT and ^14^C-acetyl coenzyme A. Reaction products were separated by SDS-PAGE and visualized by autoradiography.
(TIF)

Figure S2 **Reactivation of HIV expression from latency is suppressed in full-length 5A8 J-Lat cells activated with α-CD3/CD28 antibodies.** For the generation of J-Lat cells responsive to T cell activation, we employed a VSV-G pseudotyped HIV reporter virus containing the GFP open reading frame in place of the *Nef* gene (HIV-R7/Env-/GFP) as previously described (Jordan et al., 2003, EMBO J, 22:1868-77) to infect Jurkat cells at a multiplicity of infection of 0.1 for 96 h. GFP-positive cells were sorted twice with a FACSDiva cell sorter (Becton Dickinson) and discarded. GFP-negative cells were propagated for 1 week and stimulated with plate-bound α-CD3 (10 µg/ml) and soluble α-CD28 (2 µg/ml) antibodies for 12 h. GFP-positive cells were collected and propagated without stimulation to allow silencing of LTR transcription. Single cells were seeded in 96-well plates to generate cell clones. Examination of six clones revealed that they all had the same integration site as shown by repetitive Alu-gag PCR (Liszewski et al., 2009, Methods, 47:254-60). One of these clones, 5A8, was used in these experiments. (A) Western blot analysis of LSD1 expression in 5A8 cells infected with indicated shRNAs. (B) 5A8 cells expressing indicated shRNAs were re-stimulated with α-CD3/CD28 antibodies overnight, and GFP was measured by flow cytometry. One representative experiment performed in duplicate is shown.
(TIF)

Figure S3 **LSD1/KDM1 and CoREST do not activate basal transcription at the HIV-LTR.** (A) Lentiviral vectors expressing shRNAs against LSD1 (#1), luciferase or scrambled shRNAs were infected into J-Lat A72 cells for 6 days; cells were then stimulated with TNFα (10 ng/ml) over night. The number of GFP+ cells was analyzed by flow cytometry and expressed as percent GFP+ cells as compared to luciferase-shRNA-infected A72 cells. Results (mean±SD) of two independent experiments performed in duplicate are shown. (B) Cell viability of infected cells was assessed by LIVE/DEAD Fixable Violet Dead Cell Stain Kit (Invitrogen). Violet dye-negative (thereby viable) cells were analyzed by flow cytometry analysis. (C) Whole cell lysates from shRNA-expressing cells were analyzed by western blotting using α-LSD1 and α-tubulin antibodies. (D) Lentiviral vectors expressing shRNAs against CoREST (#2) or control shRNAs were infected into J-Lat A72 cells. The same experiment as in (A) was performed. Results (mean±SD) of two independent experiments performed in duplicate are shown. (E) Propidium iodide (PI)-negative cells (marker of cell viability) were analyzed by flow cytometry. (F) Whole cell lysates from shRNA-infected A72 cells were analyzed by western blotting using α-CoREST and α-tubulin antibodies.
(TIF)

Figure S4 **Phenelzine treatment suppresses reactivation of HIV transcription from latency in additional donors.** (A) Purified resting primary CD4+ T cells were infected at a high m.o.i. with infectious HIV-NL4-3 luciferase reporter virus. Three days after infection, cells were stimulated with α-CD3 and α-CD28 antibodies in the presence of phenelzine (100, 300, or 1000 µM) or DMSO overnight followed by analysis of luciferase activity (B) Propidium iodide (PI)-negative cells of CD4+ T cells from each donor were analyzed by flow cytometry.
(TIF)

Figure S5 **No effect of phenelzine on HIV transcription in unstimulated resting primary CD4+ T cells.** (A) Purified resting primary CD4+ T cells were infected at a high m.o.i. with infectious HIV-NL4-3 luciferase reporter virus and three days after infection were treated with phenelzine (100, 300, or 1000 µM), DRB or DMSO overnight followed by analysis of luciferase activity. (B) Propidium iodide (PI)-negative cells were analyzed by flow cytometry. Results (mean±SEM) of one experiment performed in triplicate are shown.
(TIF)

Table S1 **Posttranslational modifications of conserved residues in HIV-1 Tat and their effects on HIV transcription.**
(DOC)

## References

[1] Han Y, Wind-Rotolo M, Yang HC, Siliciano JD, Siliciano RF (2007). Experimental approaches to the study of HIV-1 latency.. Nat Rev Microbiol.

[2] Noe A, Plum J, Verhofstede C (2005). The latent HIV-1 reservoir in patients undergoing HAART: an archive of pre-HAART drug resistance.. J Antimicrob Chemother.

[3] Blazkova J, Trejbalova K, Gondois-Rey F, Halfon P, Philibert P (2009). CpG methylation controls reactivation of HIV from latency.. PLoS Pathog.

[4] Kauder SE, Bosque A, Lindqvist A, Planelles V, Verdin E (2009). Epigenetic regulation of HIV-1 latency by cytosine methylation.. PLoS Pathog.

[5] Treand C, du Chene I, Bres V, Kiernan R, Benarous R (2006). Requirement for SWI/SNF chromatin-remodeling complex in Tat-mediated activation of the HIV-1 promoter.. EMBO J.

[6] Agbottah E, Deng L, Dannenberg LO, Pumfery A, Kashanchi F (2006). Effect of SWI/SNF chromatin remodeling complex on HIV-1 Tat activated transcription.. Retrovirology.

[7] Mahmoudi T, Parra M, Vries RG, Kauder SE, Verrijzer CP (2006). The SWI/SNF chromatin-remodeling complex is a cofactor for Tat transactivation of the HIV promoter.. J Biol Chem.

[8] Van Lint C, Emiliani S, Ott M, Verdin E (1996). Transcriptional activation and chromatin remodeling of the HIV-1 promoter in response to histone acetylation.. EMBO J.

[9] Imai K, Togami H, Okamoto T (2010). Involvement of histone H3 lysine 9 (H3K9) methyltransferase G9a in the maintenance of HIV-1 latency and its reactivation by BIX01294.. J Biol Chem.

[10] Ott M, Schnolzer M, Garnica J, Fischle W, Emiliani S (1999). Acetylation of the HIV-1 Tat protein by p300 is important for its transcriptional activity.. Curr Biol.

[11] Kiernan RE, Vanhulle C, Schiltz L, Adam E, Xiao H (1999). HIV-1 tat transcriptional activity is regulated by acetylation.. EMBO J.

[12] Pagans S, Pedal A, North BJ, Kaehlcke K, Marshall BL (2005). SIRT1 regulates HIV transcription via Tat deacetylation.. PLoS Biol.

[13] Bres V, Kiernan RE, Linares LK, Chable-Bessia C, Plechakova O (2003). A non-proteolytic role for ubiquitin in Tat-mediated transactivation of the HIV-1 promoter.. Nat Cell Biol.

[14] Boulanger MC, Liang C, Russell RS, Lin R, Bedford MT (2005). Methylation of Tat by PRMT6 regulates human immunodeficiency virus type 1 gene expression.. J Virol.

[15] Van Duyne R, Easley R, Wu W, Berro R, Pedati C (2008). Lysine methylation of HIV-1 Tat regulates transcriptional activity of the viral LTR.. Retrovirology.

[16] Pagans S, Kauder SE, Kaehlcke K, Sakane N, Schroeder S (2010). The Cellular lysine methyltransferase Set7/9-KMT7 binds HIV-1 TAR RNA, monomethylates the viral transactivator Tat, and enhances HIV transcription.. Cell Host Microbe.

[17] Wei P, Garber ME, Fang SM, Fischer WH, Jones KA (1998). A novel CDK9-associated C-type cyclin interacts directly with HIV-1 Tat and mediates its high-affinity, loop-specific binding to TAR RNA.. Cell.

[18] Liang C, Wainberg MA (2002). The role of Tat in HIV-1 replication: an activator and/or a suppressor?. AIDS Rev.

[19] Weinberger LS, Dar RD, Simpson ML (2008). Transient-mediated fate determination in a transcriptional circuit of HIV.. Nat Genet.

[20] Hetzer C, Dormeyer W, Schnolzer M, Ott M (2005). Decoding Tat: the biology of HIV Tat posttranslational modifications.. Microbes Infect.

[21] Hauber J, Malim MH, Cullen BR (1989). Mutational analysis of the conserved basic domain of human immunodeficiency virus tat protein.. J Virol.

[22] Col E, Caron C, Seigneurin-Berny D, Gracia J, Favier A (2001). The histone acetyltransferase, hGCN5, interacts with and acetylates the HIV transactivator, Tat.. J Biol Chem.

[23] Dorr A, Kiermer V, Pedal A, Rackwitz HR, Henklein P (2002). Transcriptional synergy between Tat and PCAF is dependent on the binding of acetylated Tat to the PCAF bromodomain.. EMBO J.

[24] Mujtaba S, He Y, Zeng L, Farooq A, Carlson JE (2002). Structural basis of lysine-acetylated HIV-1 Tat recognition by PCAF bromodomain.. Mol Cell.

[25] Kaehlcke K, Dorr A, Hetzer-Egger C, Kiermer V, Henklein P (2003). Acetylation of Tat defines a cyclinT1-independent step in HIV transactivation.. Mol Cell.

[26] Bres V, Tagami H, Peloponese JM, Loret E, Jeang KT (2002). Differential acetylation of Tat coordinates its interaction with the co-activators cyclin T1 and PCAF.. EMBO J.

[27] Ott M, Dorr A, Hetzer-Egger C, Kaehlcke K, Schnolzer M (2004). Tat acetylation: a regulatory switch between early and late phases in HIV transcription elongation.. Novartis Found Symp:.

[28] Ivanov GS, Ivanova T, Kurash J, Ivanov A, Chuikov S (2007). Methylation-acetylation interplay activates p53 in response to DNA damage.. Mol Cell Biol.

[29] Kurash JK, Lei H, Shen Q, Marston WL, Granda BW (2008). Methylation of p53 by Set7/9 mediates p53 acetylation and activity in vivo.. Mol Cell.

[30] Deng L, de la Fuente C, Fu P, Wang L, Donnelly R (2000). Acetylation of HIV-1 Tat by CBP/P300 increases transcription of integrated HIV-1 genome and enhances binding to core histones.. Virology.

[31] Jordan A, Bisgrove D, Verdin E (2003). HIV reproducibly establishes a latent infection after acute infection of T cells in vitro.. EMBO J.

[32] Pagans S, Sakane N, Schnoelzer M, Ott M (2011). Characterization of HIV Tat modifications using novel methyl-lysine-specific antibodies.. Methods.

[33] Shi YJ, Matson C, Lan F, Iwase S, Baba T (2005). Regulation of LSD1 histone demethylase activity by its associated factors.. Mol Cell.

[34] Lee MG, Wynder C, Schmidt DM, McCafferty DG, Shiekhattar R (2006). Histone H3 lysine 4 demethylation is a target of nonselective antidepressive medications.. Chem Biol.

[35] Hakimi MA, Dong Y, Lane WS, Speicher DW, Shiekhattar R (2003). A candidate X-linked mental retardation gene is a component of a new family of histone deacetylase-containing complexes.. J Biol Chem.

[36] Humphrey GW, Wang Y, Russanova VR, Hirai T, Qin J (2001). Stable histone deacetylase complexes distinguished by the presence of SANT domain proteins CoREST/kiaa0071 and Mta-L1.. J Biol Chem.

[37] You A, Tong JK, Grozinger CM, Schreiber SL (2001). CoREST is an integral component of the CoREST- human histone deacetylase complex.. Proc Natl Acad Sci U S A.

[38] Culhane JC, Wang D, Yen PM, Cole PA (2010). Comparative analysis of small molecules and histone substrate analogues as LSD1 lysine demethylase inhibitors.. J Am Chem Soc.

[39] Mimasu S, Umezawa N, Sato S, Higuchi T, Umehara T (2010). Structurally designed trans-2-phenylcyclopropylamine derivatives potently inhibit histone demethylase LSD1/KDM1.. Biochemistry.

[40] Binda C, Valente S, Romanenghi M, Pilotto S, Cirilli R (2010). Biochemical, structural, and biological evaluation of tranylcypromine derivatives as inhibitors of histone demethylases LSD1 and LSD2.. J Am Chem Soc.

[41] Swiggard WJ, Baytop C, Yu JJ, Dai J, Li C (2005). Human immunodeficiency virus type 1 can establish latent infection in resting CD4+ T cells in the absence of activating stimuli.. J Virol.

[42] Tian X, Fang J (2007). Current perspectives on histone demethylases.. Acta Biochim Biophys Sin (Shanghai).

[43] Shi Y, Lan F, Matson C, Mulligan P, Whetstine JR (2004). Histone demethylation mediated by the nuclear amine oxidase homolog LSD1.. Cell.

[44] Metzger E, Wissmann M, Yin N, Muller JM, Schneider R (2005). LSD1 demethylates repressive histone marks to promote androgen-receptor-dependent transcription.. Nature.

[45] Huang J, Sengupta R, Espejo AB, Lee MG, Dorsey JA (2007). p53 is regulated by the lysine demethylase LSD1.. Nature.

[46] Kontaki H, Talianidis I (2010). Lysine methylation regulates E2F1-induced cell death.. Mol Cell.

[47] Wang J, Hevi S, Kurash JK, Lei H, Gay F (2009). The lysine demethylase LSD1 (KDM1) is required for maintenance of global DNA methylation.. Nat Genet.

[48] Nicholson TB, Chen T (2009). LSD1 demethylates histone and non-histone proteins.. Epigenetics.

[49] Liang Y, Vogel JL, Narayanan A, Peng H, Kristie TM (2009). Inhibition of the histone demethylase LSD1 blocks alpha-herpesvirus lytic replication and reactivation from latency.. Nat Med.

[50] Marban C, Suzanne S, Dequiedt F, de Walque S, Redel L (2007). Recruitment of chromatin-modifying enzymes by CTIP2 promotes HIV-1 transcriptional silencing.. EMBO J.

[51] Zhou M, Deng L, Lacoste V, Park HU, Pumfery A (2004). Coordination of transcription factor phosphorylation and histone methylation by the P-TEFb kinase during human immunodeficiency virus type 1 transcription.. J Virol.

[52] Zhou G, Te D, Roizman B (2010). The CoREST/REST Repressor Is both Necessary and Inimical for Expression of Herpes Simplex Virus Genes.. MBio.

[53] Grskovic M, Chaivorapol C, Gaspar-Maia A, Li H, Ramalho-Santos M (2007). Systematic identification of cis-regulatory sequences active in mouse and human embryonic stem cells.. PLoS Genet.

[54] Ventura A, Meissner A, Dillon CP, McManus M, Sharp PA (2004). Cre-lox-regulated conditional RNA interference from transgenes.. Proc Natl Acad Sci U S A.

[55] Hellman NE, Spector J, Robinson J, Zuo X, Saunier S (2008). Matrix metalloproteinase 13 (MMP13) and tissue inhibitor of matrix metalloproteinase 1 (TIMP1), regulated by the MAPK pathway, are both necessary for Madin-Darby canine kidney tubulogenesis.. J Biol Chem.

[56] Gu W, Roeder RG (1997). Activation of p53 sequence-specific DNA binding by acetylation of the p53 C-terminal domain.. Cell.

[57] Roepstorff P, Fohlman J (1984). Proposal for a common nomenclature for sequence ions in mass spectra of peptides.. Biomed Mass Spectrom.

