# Supplementary figures and images for: Activation of HIV Transcription by the Viral Tat Protein Requires a Demethylation Step Mediated by Lysine-specific Demethylase 1 (LSD1/KDM1)
Source: PLoS Pathog. 2011 Aug 18;7(8):e1002184. doi: 10.1371/journal.ppat.1002184 (PMC3158049; doi:10.1371/journal.ppat.1002184)

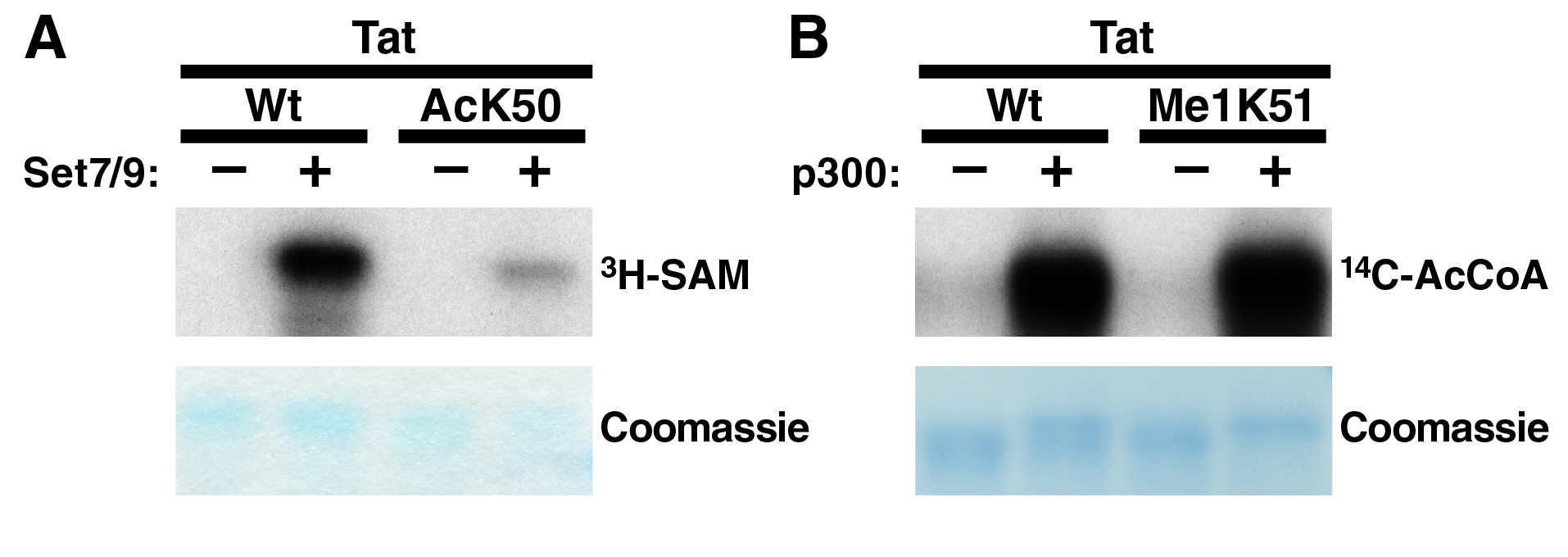

Supplement: Figure S1 — Interplay between Tat monomethylation at K51 and acetylation at K50 using synthetic Tat proteins (aa 1–72). (A) Radioactive in vitro methylation assays of synthetic Tat and K50-acetylated synthetic Tat incubated with recombinant SET7/9/KMT7 and 3H-radiolabeled S-adenosyl-L-methionine (SAM). Reaction products were separated by SDS-PAGE and visualized by autoradiography. (B) Radioactive in vitro acetylation assays of synthetic Tat and K51-methylated synthetic Tat incubated with recombinant p300-HAT and 14C-acetyl coenzyme A. Reaction products were separated by SDS-PAGE and visualized by autoradiography. (TIF) [file ppat.1002184.s001.tif]

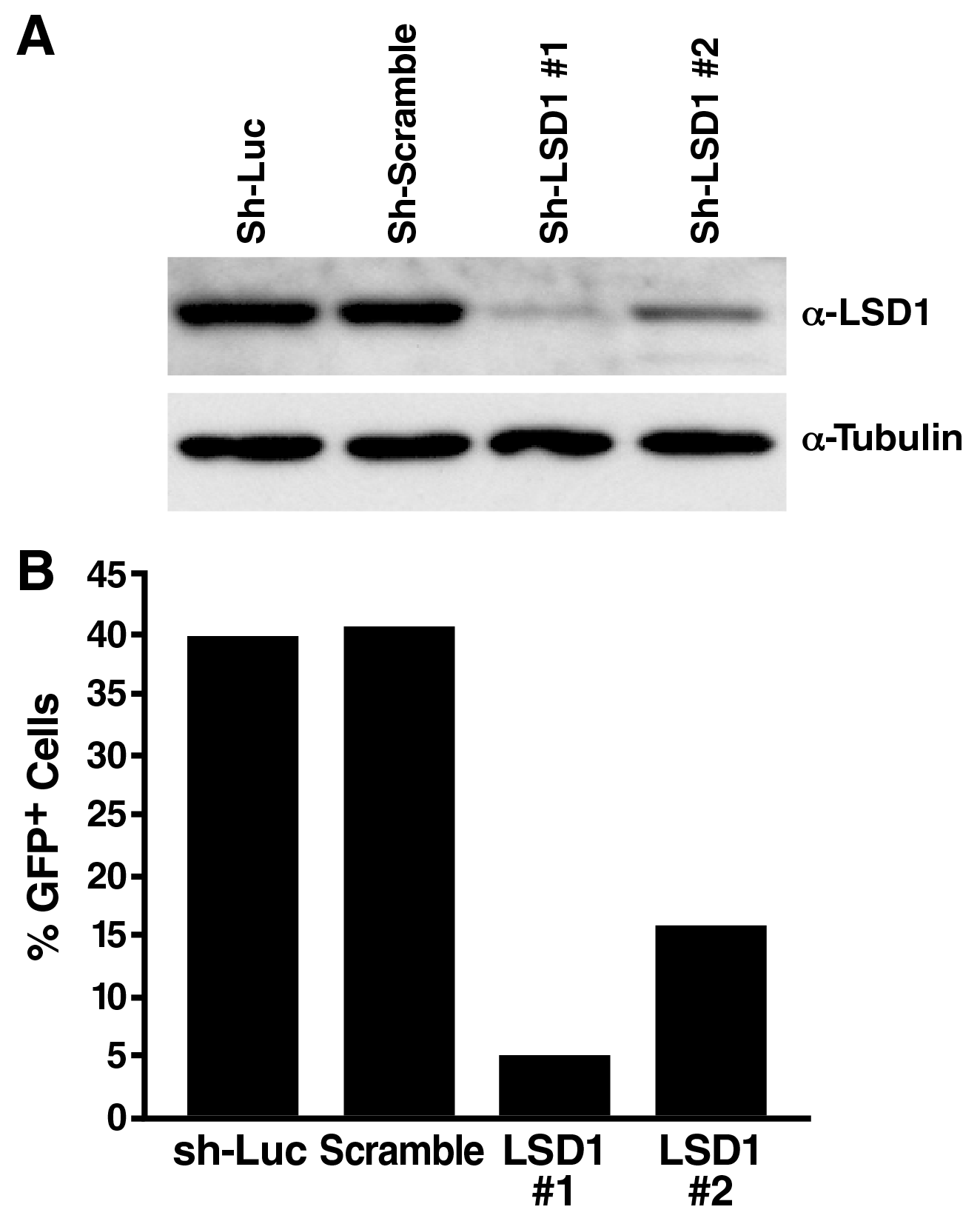

Supplement: Figure S2 — Reactivation of HIV expression from latency is suppressed in full-length 5A8 J-Lat cells activated with α-CD3/CD28 antibodies. For the generation of J-Lat cells responsive to T cell activation, we employed a VSV-G pseudotyped HIV reporter virus containing the GFP open reading frame in place of the Nef gene (HIV-R7/Env-/GFP) as previously described (Jordan et al., 2003, EMBO J, 22:1868-77) to infect Jurkat cells at a multiplicity of infection of 0.1 for 96 h. GFP-positive cells were sorted twice with a FACSDiva cell sorter (Becton Dickinson) and discarded. GFP-negative cells were propagated for 1 week and stimulated with plate-bound α-CD3 (10 µg/ml) and soluble α-CD28 (2 µg/ml) antibodies for 12 h. GFP-positive cells were collected and propagated without stimulation to allow silencing of LTR transcription. Single cells were seeded in 96-well plates to generate cell clones. Examination of six clones revealed that they all had the same integration site as shown by repetitive Alu-gag PCR (Liszewski et al., 2009, Methods, 47:254-60). One of these clones, 5A8, was used in these experiments. (A) Western blot analysis of LSD1 expression in 5A8 cells infected with indicated shRNAs. (B) 5A8 cells expressing indicated shRNAs were re-stimulated with α-CD3/CD28 antibodies overnight, and GFP was measured by flow cytometry. One representative experiment performed in duplicate is shown. (TIF) [file ppat.1002184.s002.tif]

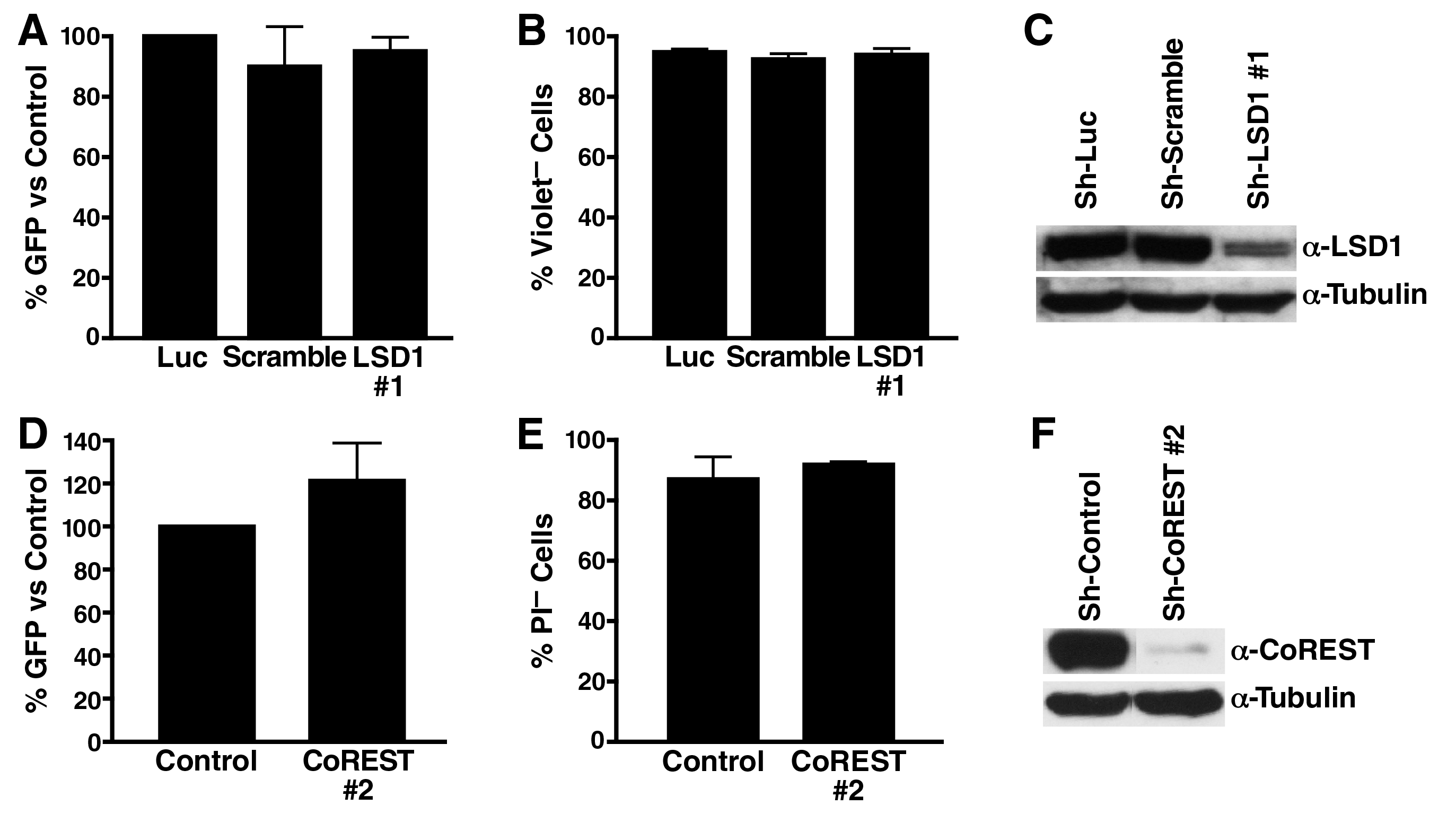

Supplement: Figure S3 — LSD1/KDM1 and CoREST do not activate basal transcription at the HIV-LTR. (A) Lentiviral vectors expressing shRNAs against LSD1 (#1), luciferase or scrambled shRNAs were infected into J-Lat A72 cells for 6 days; cells were then stimulated with TNFα (10 ng/ml) over night. The number of GFP+ cells was analyzed by flow cytometry and expressed as percent GFP+ cells as compared to luciferase-shRNA-infected A72 cells. Results (mean±SD) of two independent experiments performed in duplicate are shown. (B) Cell viability of infected cells was assessed by LIVE/DEAD Fixable Violet Dead Cell Stain Kit (Invitrogen). Violet dye-negative (thereby viable) cells were analyzed by flow cytometry analysis. (C) Whole cell lysates from shRNA-expressing cells were analyzed by western blotting using α-LSD1 and α-tubulin antibodies. (D) Lentiviral vectors expressing shRNAs against CoREST (#2) or control shRNAs were infected into J-Lat A72 cells. The same experiment as in (A) was performed. Results (mean±SD) of two independent experiments performed in duplicate are shown. (E) Propidium iodide (PI)-negative cells (marker of cell viability) were analyzed by flow cytometry. (F) Whole cell lysates from shRNA-infected A72 cells were analyzed by western blotting using α-CoREST and α-tubulin antibodies. (TIF) [file ppat.1002184.s003.tif]

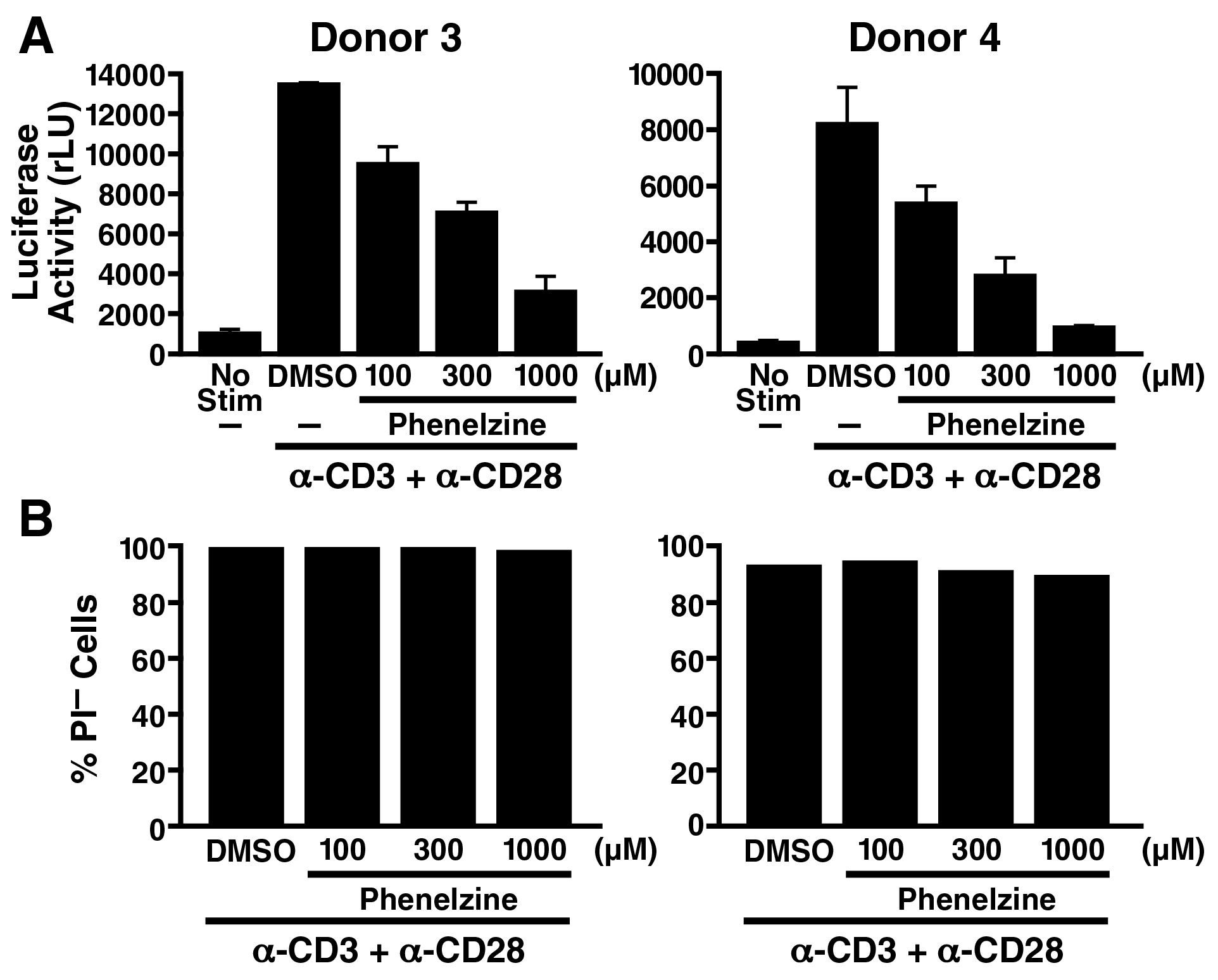

Supplement: Figure S4 — Phenelzine treatment suppresses reactivation of HIV transcription from latency in additional donors. (A) Purified resting primary CD4+ T cells were infected at a high m.o.i. with infectious HIV-NL4-3 luciferase reporter virus. Three days after infection, cells were stimulated with α-CD3 and α-CD28 antibodies in the presence of phenelzine (100, 300, or 1000 µM) or DMSO overnight followed by analysis of luciferase activity (B) Propidium iodide (PI)-negative cells of CD4+ T cells from each donor were analyzed by flow cytometry. (TIF) [file ppat.1002184.s004.tif]

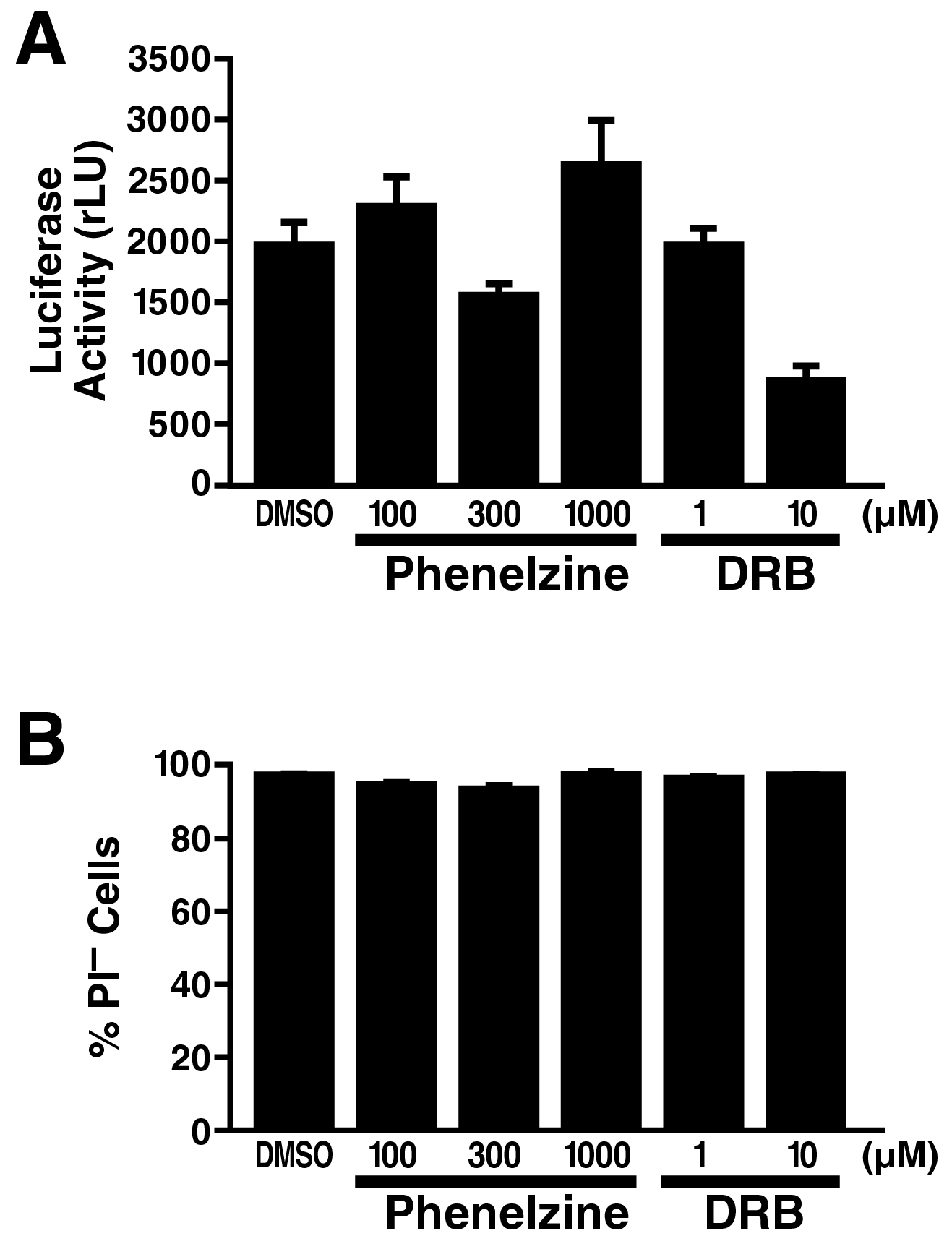

Supplement: Figure S5 — No effect of phenelzine on HIV transcription in unstimulated resting primary CD4+ T cells. (A) Purified resting primary CD4+ T cells were infected at a high m.o.i. with infectious HIV-NL4-3 luciferase reporter virus and three days after infection were treated with phenelzine (100, 300, or 1000 µM), DRB or DMSO overnight followed by analysis of luciferase activity. (B) Propidium iodide (PI)-negative cells were analyzed by flow cytometry. Results (mean±SEM) of one experiment performed in triplicate are shown. (TIF) [file ppat.1002184.s005.tif]
