# Supplementary material for: Activation of HIV Transcription by the Viral Tat Protein Requires a Demethylation Step Mediated by Lysine-specific Demethylase 1 (LSD1/KDM1)
Source: PLoS Pathog. 2011 Aug 18;7(8):e1002184. doi: 10.1371/journal.ppat.1002184 (PMC3158049; doi:10.1371/journal.ppat.1002184)
Supplement: Table S1 — Posttranslational modifications of conserved residues in HIV-1 Tat and their effects on HIV transcription. (DOC) [file ppat.1002184.s006.doc]

**Table S1. Posttranslational modifications of conserved residues in HIV-1 Tat and their effects on HIV transcription**

| **Modified Residues** | **Modification** | **Enzymes** | **HIV Transcription** | **References** |
| --- | --- | --- | --- | --- |
| S16 S46 | Phosphorylation | CDK2 | + |  |
| K28 | Acetylation | KAT2B (PCAF) | + |  |
|  | Deacetylation | HDAC6 | - |  |
| K41 K71 | Acetylation | Spontaneous | + |  |
|  |  |  |  |  |
| K50 K51 | Acetylation | KAT3B (p300) KAT2A (GCN5) | + |  |
|  | Methylation | KMT1E (SETDB1) | - |  |
| K50 | Deacetylation | SIRT1 | + |  |
| K51 | Monomethylation | KMT7 (SET7/9) | + |  |
| R52 R53 | Methylation | PRMT6 | - |  |
| K71 | Ubiquitination | MDM2 (Hdm2) | + |  |
| ? | Poly(ADP-ribosyl)ation | PARP1 | ? |  |

**References**

1. Ammosova, T., Berro, R., Jerebtsova, M., Jackson, A., Charles, S., Klase, Z., Southerland, W., Gordeuk, V. R., Kashanchi, F., and Nekhai, S. (2006) *Retrovirology* **3**, 78

2. Kiernan, R. E., Vanhulle, C., Schiltz, L., Adam, E., Xiao, H., Maudoux, F., Calomme, C., Burny, A., Nakatani, Y., Jeang, K. T., Benkirane, M., and Van Lint, C. (1999) *The EMBO J* **18**, 6106-6118

3. Huo, L., Li, D., Sun, X., Shi, X., Karna, P., Yang, W., Liu, M., Qiao, W., Aneja, R., and Zhou, J. (2011) *J Biol Chem* **286**, 9280-9286

4. Deng, L., Wang, D., de la Fuente, C., Wang, L., Li, H., Lee, C. G., Donnelly, R., Wade, J. D., Lambert, P., and Kashanchi, F. (2001) *Virology* **289**, 312-326

5. Ott, M., Schnolzer, M., Garnica, J., Fischle, W., Emiliani, S., Rackwitz, H. R., and Verdin, E. (1999) *Curr Biol* **9**, 1489-1492

6. Col, E., Caron, C., Seigneurin-Berny, D., Gracia, J., Favier, A., and Khochbin, S. (2001) *J Biol Chem* **276**, 28179-28184

7. Van Duyne, R., Easley, R., Wu, W., Berro, R., Pedati, C., Klase, Z., Kehn-Hall, K., Flynn, E. K., Symer, D. E., and Kashanchi, F. (2008) *Retrovirology* **5**, 40

8. Pagans, S., Pedal, A., North, B. J., Kaehlcke, K., Marshall, B. L., Dorr, A., Hetzer-Egger, C., Henklein, P., Frye, R., McBurney, M. W., Hruby, H., Jung, M., Verdin, E., and Ott, M. (2005) *PLoS Biol* **3**, e41

9. Pagans, S., Kauder, S. E., Kaehlcke, K., Sakane, N., Schroeder, S., Dormeyer, W., Trievel, R. C., Verdin, E., Schnolzer, M., and Ott, M. (2010) *Cell Host Microbe* **7**, 234-244

10. Boulanger, M. C., Liang, C., Russell, R. S., Lin, R., Bedford, M. T., Wainberg, M. A., and Richard, S. (2005) *J Virol* **79**, 124-131

11. Bres, V., Kiernan, R. E., Linares, L. K., Chable-Bessia, C., Plechakova, O., Treand, C., Emiliani, S., Peloponese, J. M., Jeang, K. T., Coux, O., Scheffner, M., and Benkirane, M. (2003) *Nat Cell Biol* **5**, 754-761

12. Kameoka, M., Tanaka, Y., Ota, K., Itaya, A., Yamamoto, K., and Yoshihara, K. (1999) *Biochem Biophys Res Comm* **261**, 90-94
